# Circular RNA expression and the competitive endogenous RNA network in pathological, age-related macular degeneration events: A cross-platform normalization study

**DOI:** 10.7555/JBR.37.20230010

**Published:** 2023-04-28

**Authors:** Ruxu Sun, Hongjing Zhu, Ying Wang, Jianan Wang, Chao Jiang, Qiuchen Cao, Yeran Zhang, Yichen Zhang, Songtao Yuan, Qinghuai Liu

**Affiliations:** Department of Ophthalmology, the First Affiliated Hospital of Nanjing Medical University, Nanjing Medical University, Nanjing, Jiangsu 211166, China

**Keywords:** age-related macular degeneration, retinal pigment epithelium, circular RNA, bioinformatics analysis, competing endogenous RNA

## Abstract

Age-related macular degeneration (AMD) causes irreversible blindness in people aged over 50 worldwide. The dysfunction of the retinal pigment epithelium is the primary cause of atrophic AMD. In the current study, we used the ComBat and Training Distribution Matching method to integrate data obtained from the Gene Expression Omnibus database. We analyzed the integrated sequencing data by the Gene Set Enrichment Analysis. Peroxisome and tumor necrosis factor-α (TNF-α) signaling and nuclear factor kappa B (NF-κB) were among the top 10 pathways, and thus we selected them to construct AMD cell models to identify differentially expressed circular RNAs (circRNAs). We then constructed a competing endogenous RNA network, which is related to differentially expressed circRNAs. This network included seven circRNAs, 15 microRNAs, and 82 mRNAs. The Kyoto Encyclopedia of Genes and Genomes analysis of mRNAs in this network showed that the hypoxia-inducible factor-1 (HIF-1) signaling pathway was a common downstream event. The results of the current study may provide insights into the pathological processes of atrophic AMD.

## Introduction

Age-related macular degeneration (AMD) is a progressive disease characterized by central detailed vision damage and is the leading cause of irreversible blindness in people aged over 50 worldwide. The number of people suffering from AMD is expected to reach 288 million by the year 2040, which increases the pressure on human finite resources. AMD can be categorized into two forms, exudative and atrophic, based on choroidal neovascularization ^[ 1] ^. Currently, due to the deep understanding of the mechanisms underlying neovascularization, anti-vascular endothelial growth factor (VEGF) injection has been developed as the most effective treatment for the wet AMD. However, there is no proven treatment for atrophic AMD, and therefore, studies focusing on the pathological process of the dry AMD are needed.

Retinal pigment epithelial (RPE) consists of hexagonal and polarized cells localized between the photoreceptors and Bruch's membrane. These cells are crucial for maintaining retinal homeostasis, and depletion of their functions can cause multiple retinal degeneration diseases, including AMD. Oxidative stress and inflammation are two profound pathological factors contributing to RPE dysfunction ^[ 2] ^. Oxidative stress, supported by environmental and genetic factors, has long been considered a contributor to RPE dysfunction in AMD. Moreover, smoking is the strongest risk factor for AMD, as it can intensify the high-oxygen environment in the RPE lives ^[ 1] ^. In addition, variants of genes encoding oxidative stress-related proteins are associated with the risk of AMD. For example, people with variants in NADH dehydrogenase subunits, mitochondrial MTND2*LHON4917G and superoxide dismutase 2 (SOD2) are more likely to suffer from AMD, compared with those who do not have such variants ^[ 3– 4] ^. Apart from oxidative stress, the high rate of blood flow in the macular choriocapillaris may expose RPE cells to a high level of circulating inflammatory mediators. The increased concentrations of soluble tumor necrosis factor (TNF) receptor Ⅱ (TNFR2), chemokine (C-X-C motif) ligand 10 (CXCL10), and interleukins ( *i.e.*, IL-6, IL-7 and IL-17) ^[ 5]^ in the serum have been discovered in AMD patients. Additionally, the increased TNF-α level in blood monocytes is also associated with AMD ^[ 6] ^.

Circular RNAs (circRNAs) are a novel class of noncoding RNAs that have a covalently linked circular structure through reverse splicing, and are more stable, compared to long non-coding RNAs (lncRNAs) ^[ 7] ^. They play pivotal roles in pathological processes, including tumorigenesis, cell differentiation, and neurodegeneration. Some circRNAs can also be important epigenetic regulators in diseases. For example, circRNAs can function as competing endogenous RNAs to regulate gene expression at the post-transcriptional level. We have also previously identified circNR3C1 that functions as a competitive endogenous RNA to block the interaction between miR-382-5p and PTEN to prevent AMD progression ^[ 8] ^. However, circRNA expression profiling of the dysregulated RPE in AMD has not been adequately investigated.

Transcriptomic data provide the opportunity to systemically study pathological processes and biological pathways. However, the relatively small sample size limits statistical power to identify disease signatures ^[ 9] ^. Another problem is the difficulty of combining RNA-seq and microarray data. Cross-platform normalization of microarray and RNA-seq integrates sequencing data from different platforms with sufficient distribution similarities. Among all these methods, Training Distribution Matching (TDM), an approach transforming the distribution of RNA-seq data to microarray data based on machine learning, has the best performance ^[ 10] ^.

## Materials and methods

**Figure 1:**
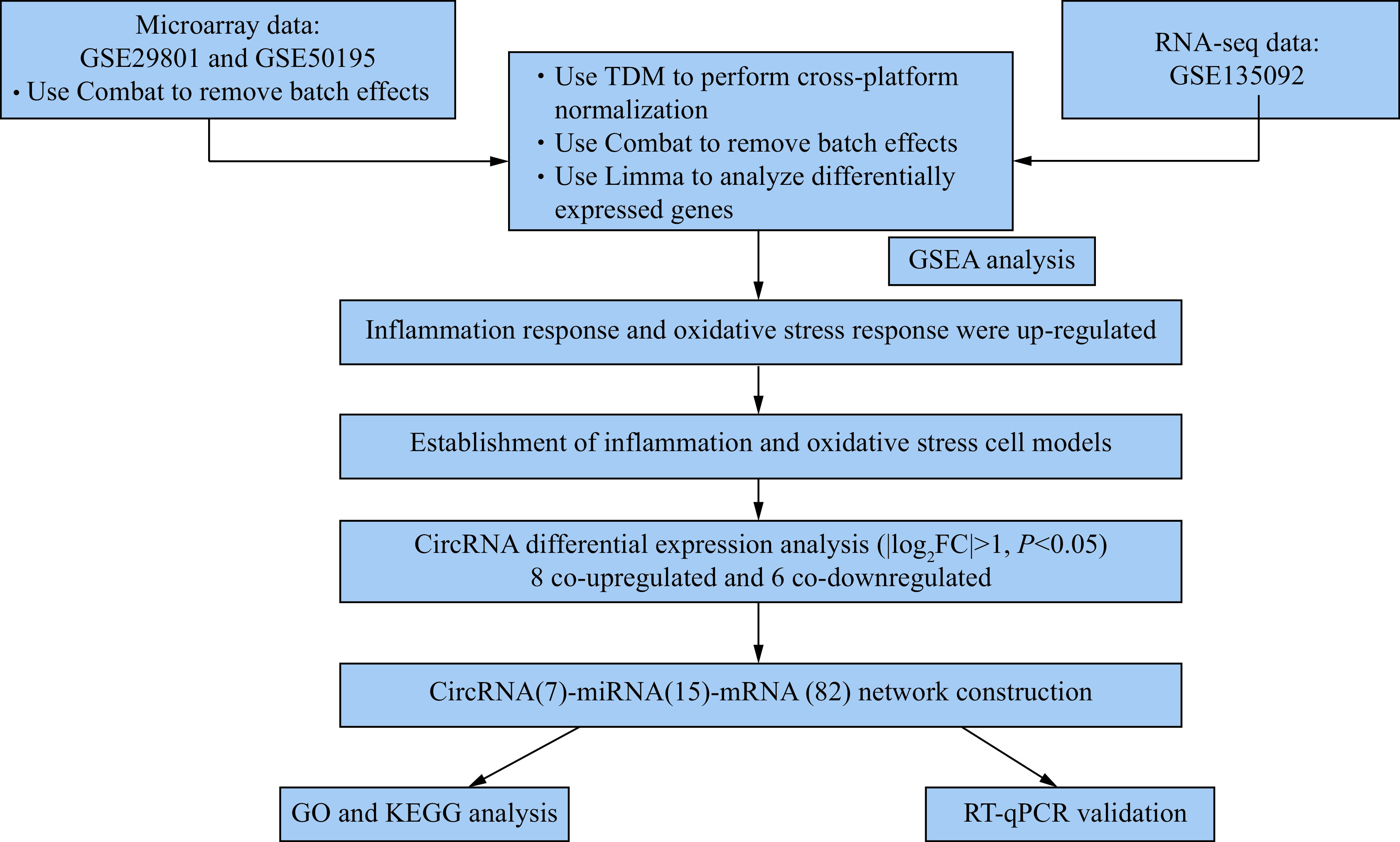
The flowchart of cross-platform integration and circRNA-miRNA-mRNA network construction.

The current study aimed to investigate the role of circRNAs and their associated competitive endogenous RNA network in pathological AMD events. TDM was used to integrate three public datasets from the Gene Expression Omnibus (GEO) database. Gene expression patterns were explored by Gene Set Enrichment Analysis (GSEA), and several new pathological processes were identified. TNF-α signaling *via* nuclear factor kappa B (NF-κB) and oxidative stress were among the top 10 pathways. Because dysfunctional RPE is the initial trigger of AMD, RPE cell models were constructed accordingly. In both cell models, we performed circRNA expression profiling and constructed a competitive endogenous RNA network to explore the affected pathological pathways. Furthermore, the verification of differentially expressed circRNAs in cell models was performed. A flowchart of the current study is shown in ***Fig. 1***.

### Data collection and preprocessing

Three human AMD transcriptome datasets (GSE29801, GSE50195, and GSE135092) in the GEO database were included. High-throughput sequencing results from GSE135092 were derived from an RNA-seq study performed under the Illumina platform ^[ 11] ^. Microarray data from GSE50195 were derived from a study using Affymetrix Human Exon ST 1.0 arrays, and data extracted from GSE29801 were obtained from a study using the Agilent G4112F array ^[ 12] ^. To eliminate low-quality samples, we used the array QualityMetrics package in R ^[ 13] ^. The individual array quality section was considered. Samples with Da > 0.15 were identified as outliers ^[ 13] ^.

### Integration and analysis of sequencing data from the GEO database

To reduce the impact of confounding factors derived from batch effects and cross-platform differences, we first used ComBat to combine two microarray datasets from GSE29801 and GSE50195, and then used TDM to integrate RNA-seq data from GSE135092 to integrate microarray data. Principal component analysis (PCA) was used to reveal sample heterogeneity. After integration, the Limma package was used to identify differentially expressed genes in the merged dataset ^[ 14] ^.

#### Gene set enrichment analysis

To identify biological changes in AMD, we used GSEA to analyze enriched concepts in clinical samples. There are eight gene sets in the Molecular Signatures Database (MSigDB) that providing gene set annotations for GSEA. We focused on the well-defined and coherently expressed signatures, called the Hallmark Gene Set. A threshold of *P* < 0.05 was established to determine the altered biological processes.

#### Protein-protein interaction (PPI) analysis

PPI network of differentially expressed mRNAs was conducted using STRING (version 11.5). Nodes with interaction scores of more than 0.4 were included, and disconnected nodes were excluded from the network. Genes with more than five nodes were shown in the network.

### Cell culture and treatments

ARPE-19 cells were purchased from American Type Culture Collection and cultured using Dulbecco's modified Eagle's/F12 medium, supplemented with 10% fetal bovine serum (Invitrogen, Carlsbad, CA, USA) and 1% penicillin/streptomycin (Gibco, Grand Island, NY, USA) at 37 ℃, 5% CO _2_. The concentration and duration of treatments were conducted as previously described ^[ 15] ^. In brief, cells were incubated in 200 μmol/L H _2_O _2_ (Sigma, St. Louis, MO, USA) for 24 h or 100 ng/mL TNF-α (Novoprotein, Shanghai, China) for 48 h before collecting for RNA-seq.

### CircRNA identification

#### Total RNA isolation

Total RNA from ARPE19 cells treated with H _2_O _2_ or TNF-α was extracted using TRIzol (Invitrogen) and subsequently detected by Nano-Drop ND-1000 spectrophotometer (Nano-Drop Technologies, Wilmington, NC, USA) to determine the concentration and purity of total RNA from each sample.

#### CircRNA library construction

The integrity of RNA extracted as mentioned above was assessed by Bioanalyzer 2100 (Agilent, Palo Alto, USA) with an RIN number >7.0, and further confirmed by electrophoresis with denaturing agarose gel electrophoresis. Ribosomal RNA in total RNA (3 μg) was removed using the Epicentre Ribo-Zero Gold Kit (Illumina, San Diego, CA, USA). The linear RNA was digested using Epicentre Ribonuclease R (Illumina).

After purification, the remaining RNA was fragmented using Magnesium RNA Fragmentation Module (NEB, Ipswich, MA, USA) at 94 ℃ for 5 to 7 mins. After that, the cleaved RNA fragments were reverse-transcribed to create the cDNA using SuperScript Ⅱ Reverse Transcriptase (Invitrogen). *E*. *coli* DNA polymerase Ⅰ (NEB), Rnase H (NEB), and dUTP Solution (Thermo Fisher, Waltham, MA, USA) were next used to synthesize U-labeled second-stranded DNAs.

An A-base was added to the blunt ends of each strand, preparing them for ligation to the indexed adapters. Each adapter contained a T-base overhang for ligating the adapter to the A-tailed fragmented DNA. Single- or dual-index adapters were ligated to the fragments, and size selection was performed using AMPureXP beads (Beckman Coulter, CA, USA). After treatment with the heat-labile UDG enzyme (NEB), the ligated products were amplified with PCR. The average insert size for the final cDNA library was 300 (± 50) bp. Finally, the 2 × 150-bp paired-end sequencing was performed (PE150) on an llumine Novaseq 6000 (LC-Bio Technology Co., Hangzhou, China) following the manufacturer's protocol.

#### Prediction of circRNAs

We used CIRCExplorer to *de novo* assemble the mapped reads to circRNAs. The back-splicing reads were then identified in unmapped reads by tophat-fusion and CIRCExplorer. CircRNA expressions from different samples or groups were calculated by SRPBM = (number of back-spliced junction reads)/(number of mapped reads) × 1000000000.

### Identification of differentially expressed mRNAs, microRNAs and circRNAs

The expressions of mRNAs, microRNAs and circRNAs were analyzed using Stringtie algorithm with differential expression annotated by Deseq2 software.

### Construction of circRNA-miRNA-mRNA network

CircRNA-miRNA interactions were predicted using the CircInteractom ( https://circinteractome.nia.nih.gov) and Circbank ( http://www.circbank.cn/index.html) databases to detect potential binding sites within genomic sequences as previously described ^[ 16] ^. TargetScan ( https://www.targetscan.org/vert_72/), miRanda ( http://www.microrna.org/), and miRDB ( http://www.mirdb.org/) were used to construct miRNA-mRNA pairs as previously described ^[ 17] ^. The predicted genes that were consistent with differentially expressed mRNAs in two cell models were retained as target genes in the network. The Cytoscape software (version 3.6.1, USA) was used to visualize the circRNA-miRNA-mRNA network.

### Kyoto Encyclopedia of Genes and Genomes (KEGG) and Gene Ontology (GO) analyses

GO is a major initiative that focuses on the functions of a cluster of genes to provide an overview of differential genes. KEGG is composed of disease and metabolic pathway enrichment ^[ 18] ^. Because mRNAs in the competitive endogenous RNA network do not have fold change (FC) information and cannot be analyzed with GSEA, the GO and KEGG databases were used to annotate gene functions. The GO and KEGG enrichment analyses of genes were conducted separately using the enrichGO and enrichKEGG functions in the clusterProfiler R package ^[ 19] ^. The GO or KEGG terms fulfilling the condition of *P* < 0.05 were defined as significantly enriched GO/KEGG terms.

### Real-time quantitative PCR validation of differentially expressed circRNAs and mRNAs

CircRNAs and mRNAs in the competitive endogenous RNA network were randomly selected from the RNA-seq data for validation. Cells treated with H _2_O _2_ or TNF-α were used to validate the expressions of circRNAs and mRNAs by real-time quantitative PCR (RT-qPCR). RT-qPCR was conducted according to the previously described instructions ^[ 8] ^. Total RNA was isolated as mentioned above. Reverse transcriptase PCR was performed with 500 ng total RNA using PrimeScript RT kit (Takara, Otsu, Shiga, Japan), and RT-qPCR was subsequently performed using StepOne Plus real-time PCR system (Applied Biosystems, Waltham, MA, USA). The 18S and GAPDH were used as the internal references for circRNAs and mRNAs, respectively. The primer sequences used for RT-qPCR are listed in ***Supplementary Table 1*** (available online).

### Immunoblotting

Cells from distinct groups were collected in lysis buffer (Beyotime Biotechnology, Shanghai, China) supplemented with protease inhibitors cocktail (Roche, Basel, Switzerland) for protein extraction. The isolated proteins were then segregated with gel electrophoresis and transferred onto a polyvinylidene fluoride membrane (Roche). Then, the membranes were incubated in distinct antibodies (anti-ZO-1 antibody [Cat. #61-7300, Invitrogen], anti-β-catenin antibody [Cat. #ab32572, Abcam, Cambridge, UK] or anti-β-actin antibody [Cat. #93473s, Cell Signaling Technology, Danvers, MA, USA]) at 4 ℃ overnight, followed by HRP-conjugated anti-Rabbit antibody incubation (Cat. #7074s, Cell Signaling Technology) at room temperature for 2 h. Tanon-5200Multi Chemiluminescent Imaging System (Tanon Science & Technology Co., Ltd. Shanghai, China) was used to develop the blots.

**Table 1:** Basic characteristics of 13 co-regulated circRNAs

circBase ID ^*^	Type	Gene name	Strand	Chromosome	Genomic length (nt)	Spliced sequence length (nt)
hsa_circ_0001400	Up	*RELL1*	−	37633006–37640126	7120	434
hsa_circ_0003353	Down	*TFPI*	−	188348850–188368497	19 647	630
hsa_circ_0004777	Down	SOX13	+	204082042–204083733	1691	419
hsa_circ_0006847	Down	*ASPHD1*	+	29916172–29917280	1108	286
hsa_circ_0095556	Down	*NAV2*	+	19998292–20005793	7501	7501
hsa_circ_0111274	Up	*PAPPA2*	+	176524542–176526377	1835	1835
hsa_circ_0128921	Up	*NIPBL*	+	36982266–36986403	4137	4137
hsa_circ_0007345	Down	*DLG1*	−	196812450–196846401	33 951	673
hsa_circ_0001658	Down	*ARID1B*	+	157357968–157406039	48 071	48 071
hsa_circ_0005114	Down	*RIMS2*	+	105080739–105161076	80 337	80 337
hsa_circ_0000339	Up	*RAB6A*	−	73418464–73429763	11 299	312
hsa_circ_0101802	Up	*PNN*	+	39648294–39648666	372	295
hsa_circ_0001173	Up	*VAPB*	+	57014000–57016139	2139	258
^*^One cirRNA could not be identified in the known circRNA databases, and thus it isn't shown. Abbreviation: nt, nucleotide.						

### Immunofluorescence (IF)

IF was conducted as previously described ^[ 8] ^. Briefly, ARPE-19 cells were seeded into 8-well chamber slides (Millipore, MA, USA) and treated with either TNF-α or H _2_O _2_. After treatment, cells were harvested and incubated in diluted primary antibodies at 4 ℃ overnight and corresponding fluorescence-conjugated secondary antibodies at room temperature for 2 h. Cell nuclei were stained with DAPI (Beyotime Biotechnology). Images were then captured using the LSM 510 confocal microscope (Zeiss, Jena, Germany).

### Statistical analysis

GraphPad Prism (version 9.0) was used to analyze data and construct figures. The Mann-Whitney's test was used to compare the relative intensity of IF between two different groups. For RT-qPCR and immunoblotting analysis, Student's *t*-test was used. Data were presented as means with standard errors of the mean, and *P* < 0.05 was considered as statistically significant.

## Results

### Cross-platform integration of GEO datasets and bioinformatics analysis

**Figure 2:**
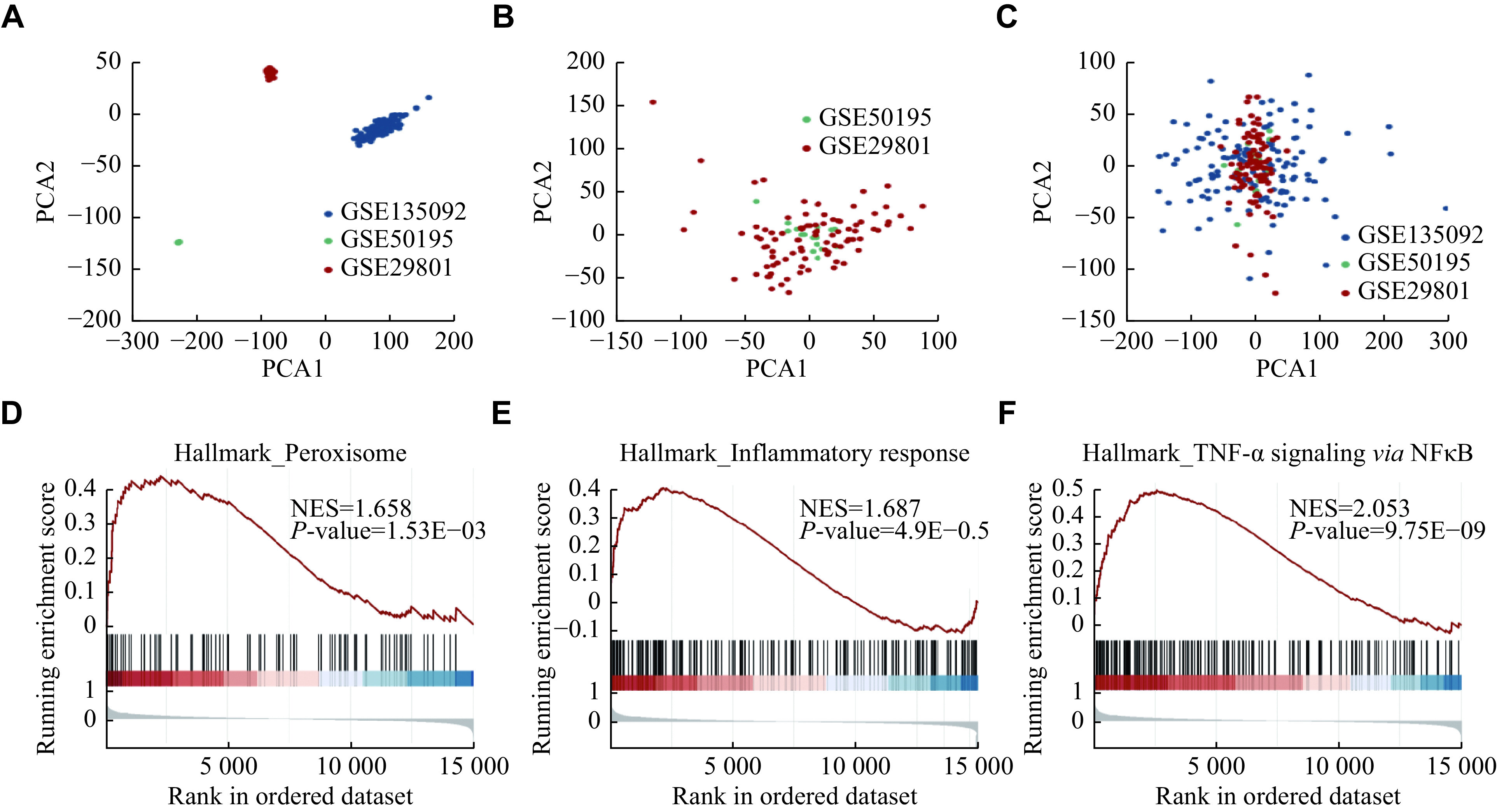
Cross-platform integration of Gene Expression Omnibus datasets and bioinformatic analysis.

To identify gene expressions in AMD, we complied an AMD meta-cohort from three AMD cohorts. Because dry AMD mainly affects the RPE-choroid complex in the macular, only macular AMD RPE/choroid samples were included, and wet AMD samples were excluded. In all, 162 normal samples and 59 AMD samples were recruited. There were 67 males and 95 females in the control group, and 28 males and 31 females in the AMD group. We also implemented a quality control method to assess the quality of array samples and eliminate outliers. The samples included in the current study were all qualified. The results of quality control are shown in ***Supplementary Fig. 1 ***. We constructed a PCA to measure sample heterogeneity among the three cohorts ( ***Fig. 2A***), and then used ComBat to eliminate batch effects and TDM to integrate microarray and RNA-seq results. After integration, there was no obvious difference between the sample clusters ( ***Fig. 2B*** and ***2C***). The top 50 upregulated and downregulated genes are listed in ***Supplementary Table 2*** (available online).

Compared with KEGG and GO analyses that only focus on genes above a certain threshold, GSEA considers all genes and provides a more accurate result. Therefore, we used GSEA to identify the dysregulated biological processes in AMD samples from the GEO database. Peroxisome and inflammation responses were activated in AMD ( ***Fig. 2D*** and ***2E***), indicating the increased levels of reactive oxygen species (ROS) and inflammatory cytokines. Additionally, TNF-α signaling *via* NF-κB, a specific inflammatory process, ranked the first in AMD ( ***Fig. 2F***). The bioinformatic results of GSEA are listed in ***>Supplementary Table 3*** (available online).

**Figure 3:**
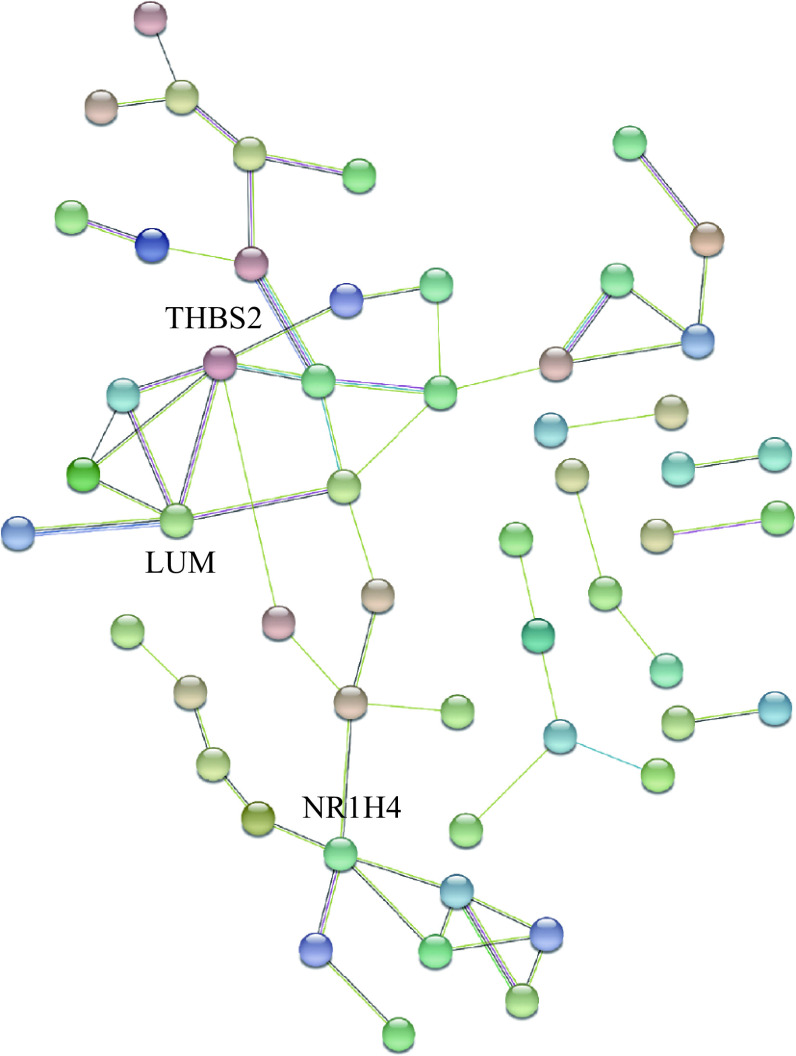
Protein-protein interaction analysis of differentially expressed genes in integrated sequencing data of AMD.

To reveal core regulator genes in AMD, we turned to the STRING database to predict the protein interactions among differentially expressed genes. mRNAs with more than five connection nodes, including thrombospondin 2 ( *THBS2*), lumican ( *LUM*), and nuclear receptor subfamily 1 group H member 4 ( *NR1H4*), were identified as key genes ( ***Fig. 3***) and discussed in the Discussion part.

### Construction and validation of inflammation and oxidative stress cell models

**Figure 4:**
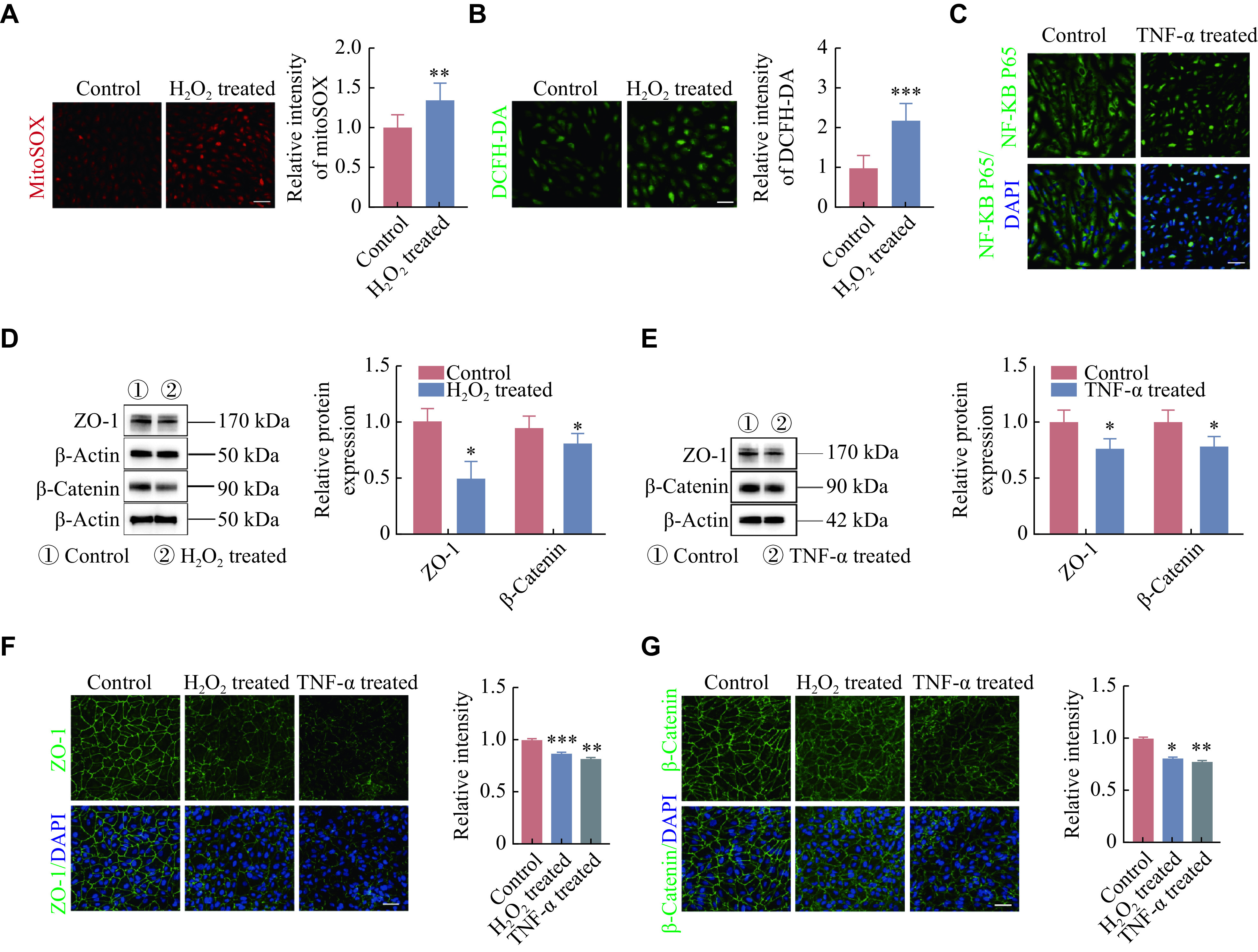
Construction of inflammation and oxidative stress cell models.

Based on our data, oxidative stress and TNF-α signaling *via* NF-κB were the most upregulated in AMD. Therefore, we constructed two cell models accordingly for further analysis. IF was performed to detect ROS levels, and the results showed that the ROS generation in mitochondria was significantly increased in RPE cells treated with H _2_O _2_, compared with the control group ( ***Fig. 4A***). Moreover, H _2_O _2_ treatment increased total ROS levels in RPE cells ( ***Fig. 4B***). Furthermore, we used IF to detect the localization of NF-κB p65 to see if TNF-α signaling activated this transcription factor. Cells treated with TNF-α presented nucleus translocation of NF-κB ( ***Fig. 4C***). These data indicated that the two cell models were successfully established.

We next set out to detect the phenotype of dry AMD in these cell models. RPE cells undergo dedifferentiation or depolarization during the pathology of AMD. During this process, RPE cells gradually lost tight junctions essential for building RPE polarity. We therefore investigated whether oxidative stress and inflammation could induce RPE depolarization. As revealed by immunoblotting, RPE cells exhibited a decreased level of ZO-1, a tight junction that forms at the RPE cell membrane, after being treated with H _2_O _2_ or TNF-α ( ***Fig. 4D*** and ***4E***). Consistently, the expression of β-catenin, a protein that binds with epithelial cadherins to generate adherences junctions, was also decreased in RPE after treatment with H _2_O _2_ or TNF-α ( ***Fig. 4D*** and ***4E***). Additionally, RPE cells treated with TNF-α showed wavy cell borders, which was also reported in RPE cells with lower RPE characteristics ^[ 20] ^. IF further confirmed the results of immunoblotting ( ***Fig. 4F*** and ***4G***). Based on these findings, cells treated with H _2_O _2_ or TNF-α showed reduced RPE markers, indicating the phenotype of AMD, and thus, these cells could be used as cell models of this disease.

### Identification of differentially expressed circRNAs in AMD cell models

**Figure 5:**
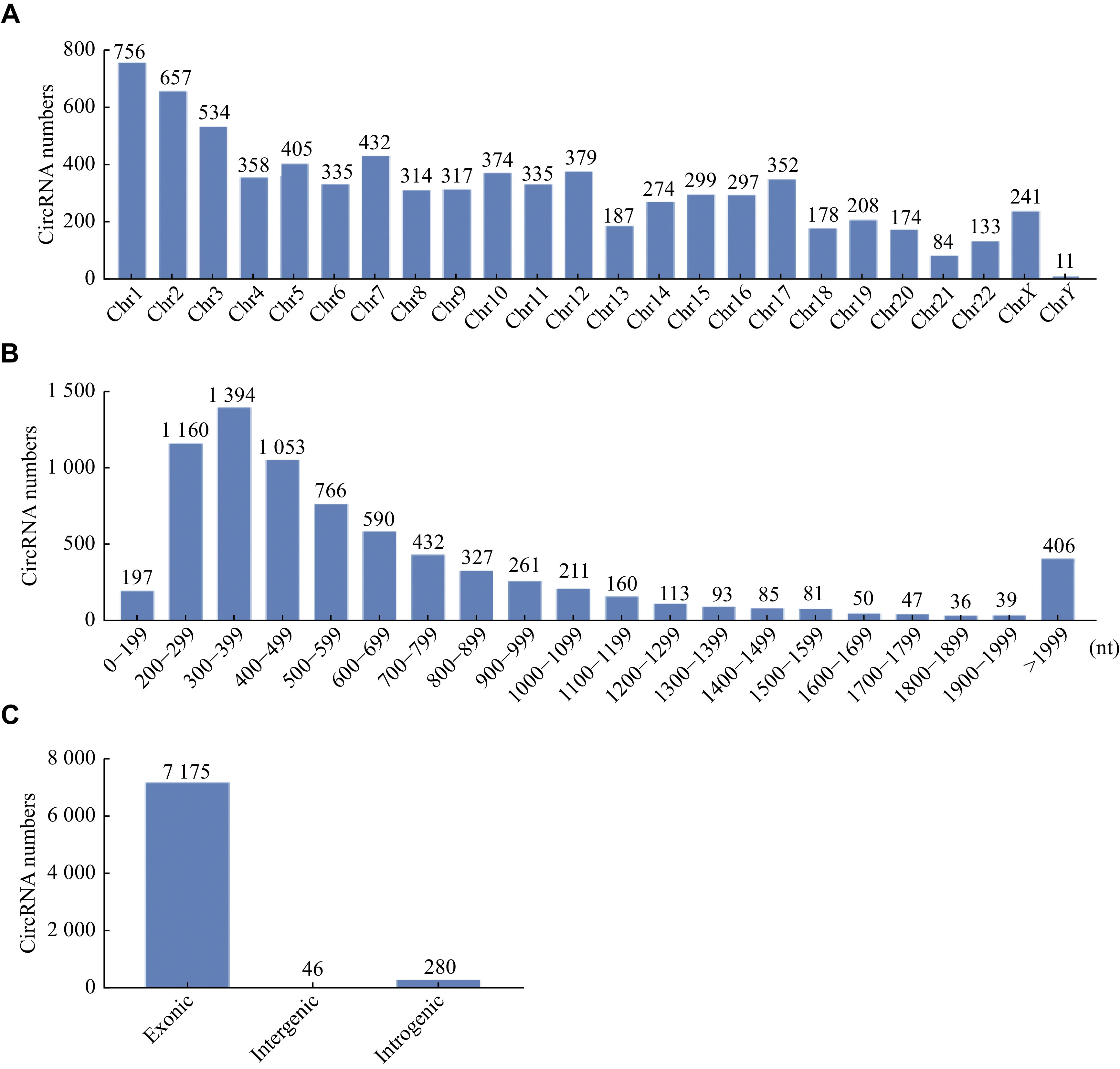
Characterization of identified circRNAs.

For the oxidative stress and inflammation models of RPE, a total of 7501 circRNAs were identified. These circRNAs were distributed among the entire genome, with the majority of them localized in chromosomes 1 and 2 ( ***Fig. 5A***). The length of the identified circRNAs ranged from less than 200 to more than 1999 nucleotides ( ***Fig. 5B***). The majority of circRNAs were less than 2000 nucleotides in length (7095/7501; 94.6%). The classification of circRNAs was analyzed ( ***Fig. 5C***). These circRNAs compromised 7175 extronic, 46 intergenic, and 280 intergenic regions.

**Figure 6:**
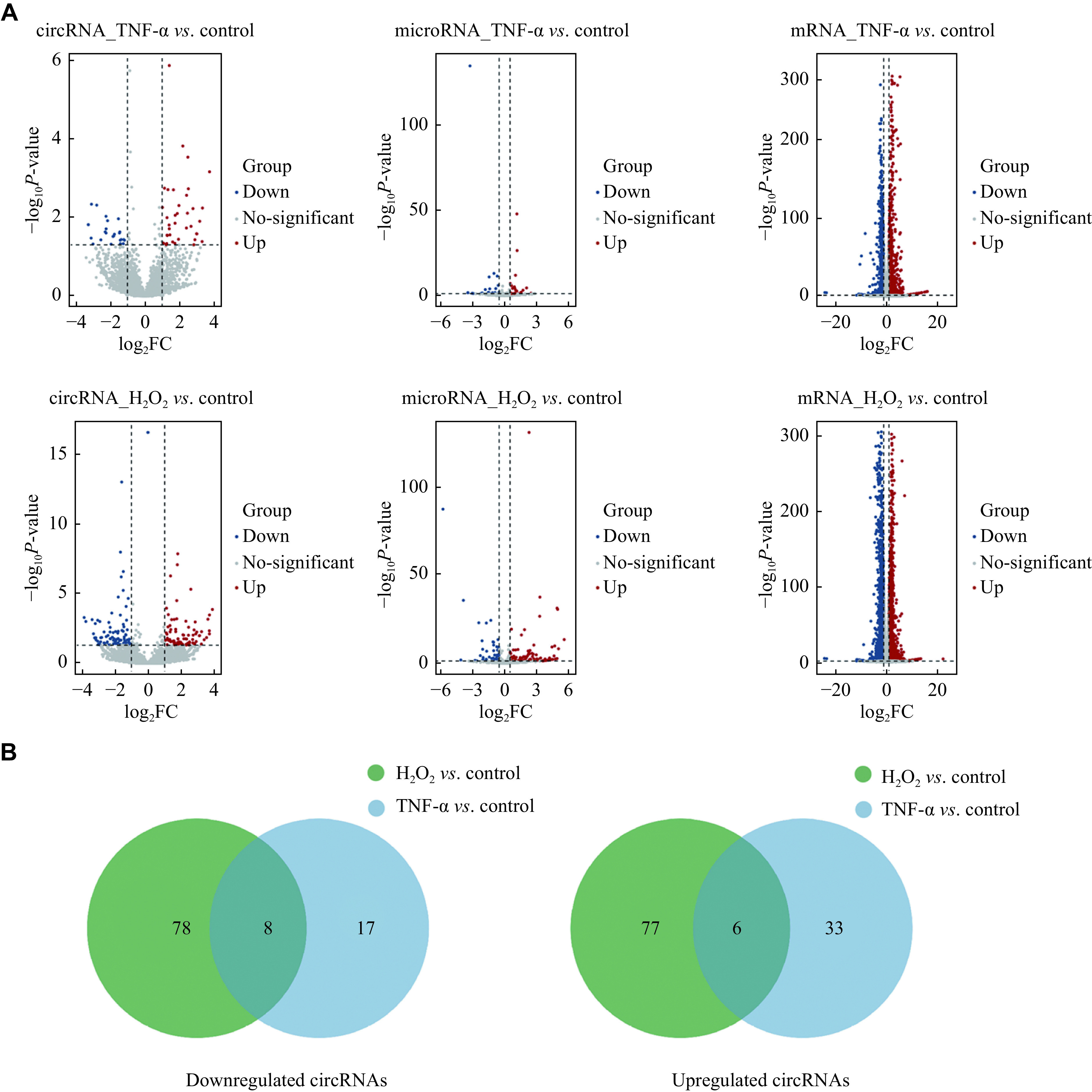
An overview of the expression profiling of RNAs in oxidative stress and inflammation cell models.

The sequencing data showed that there were 83 upregulated and 86 downregulated circRNAs in the oxidative stress model and 39 upregulated and 25 downregulated circRNAs in the inflammatory cell model (|log _2_FC| > 1, *P* < 0.05). Regarding miRNAs, 95 were upregulated and 64 were downregulated in the oxidative stress model, while 19 were upregulated and 24 were downregulated in the inflammatory cell model (|log _2_FC| > 0.5, *P* < 0.05). In terms of mRNAs, there were 3285 upregulated and 2419 downregulated mRNAs in the oxidative stress model. Additionally, 1317 upregulated and 1110 downregulated mRNAs were found in the inflammatory cell model (|log _2_FC| > 1, *P* < 0.05). Volcano plots were constructed to provide an overview of the differentially expressed RNAs ( ***Fig. 6A***). Based on the GSEA results of the sequencing data from clinical AMD samples, both oxidative stress and TNF-α induced inflammation played important roles in this pathological process. As a result, circRNAs with the same expression pattern in these two models were chosen for further analysis. Altogether, there were eight co-downregulated and six co-upregulated circRNAs in the two AMD models ( ***Table 1*** and ***Fig. 6B***).

### Construction of the circRNA-miRNA-mRNA regulatory network

**Figure 7:**
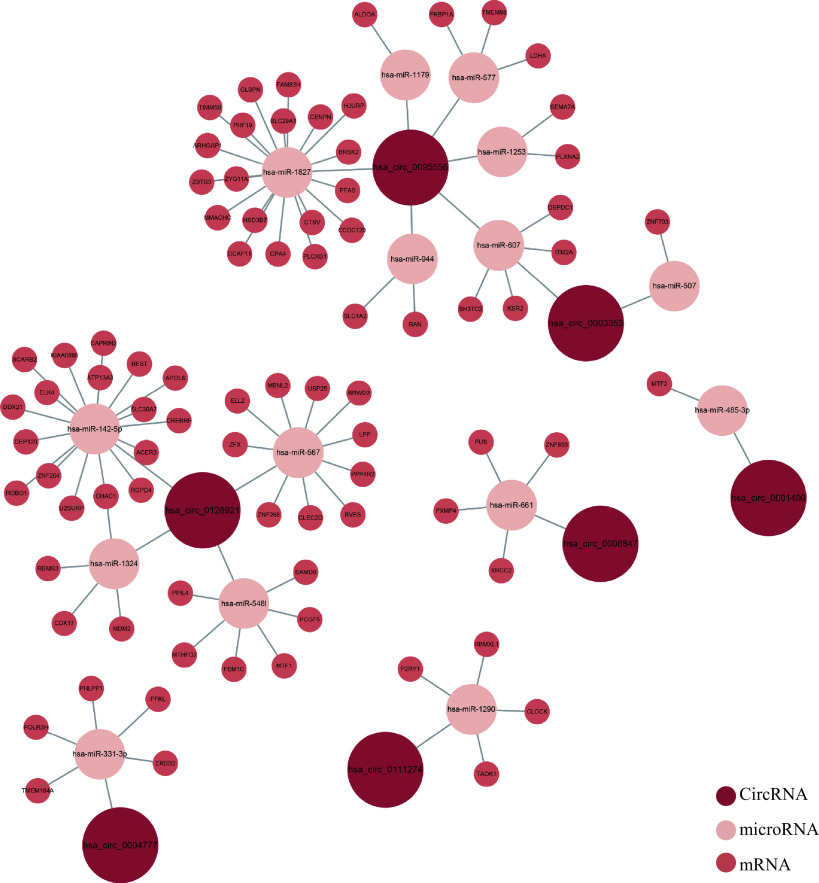
Differentially expressed circRNAs mediated competitive endogenous RNA network.

After identifying the differentially expressed circRNAs, we used online tools to predict possible miRNAs for these circRNAs. Among these co-regulated circRNAs, one could not be identified in the known circRNA databases, and six did not have common target miRNAs in the CircInteractome and Circbank databases. These seven circRNAs were eliminated from the circRNA-miRNA-mRNA network. After all, 15 miRNAs were predicted as targets of the seven circRNAs. For the identification of miRNA-mRNA pairs, miRanda, miRDB and TargetScan were used to predict target genes of the 15 miRNAs. The predicted mRNAs that overlapped with co-differentially expressed genes in the two cell models were accepted as target mRNAs. Based on these results, a competitive endogenous RNA network was constructed ( ***Fig. 7***).

**Figure 8:**
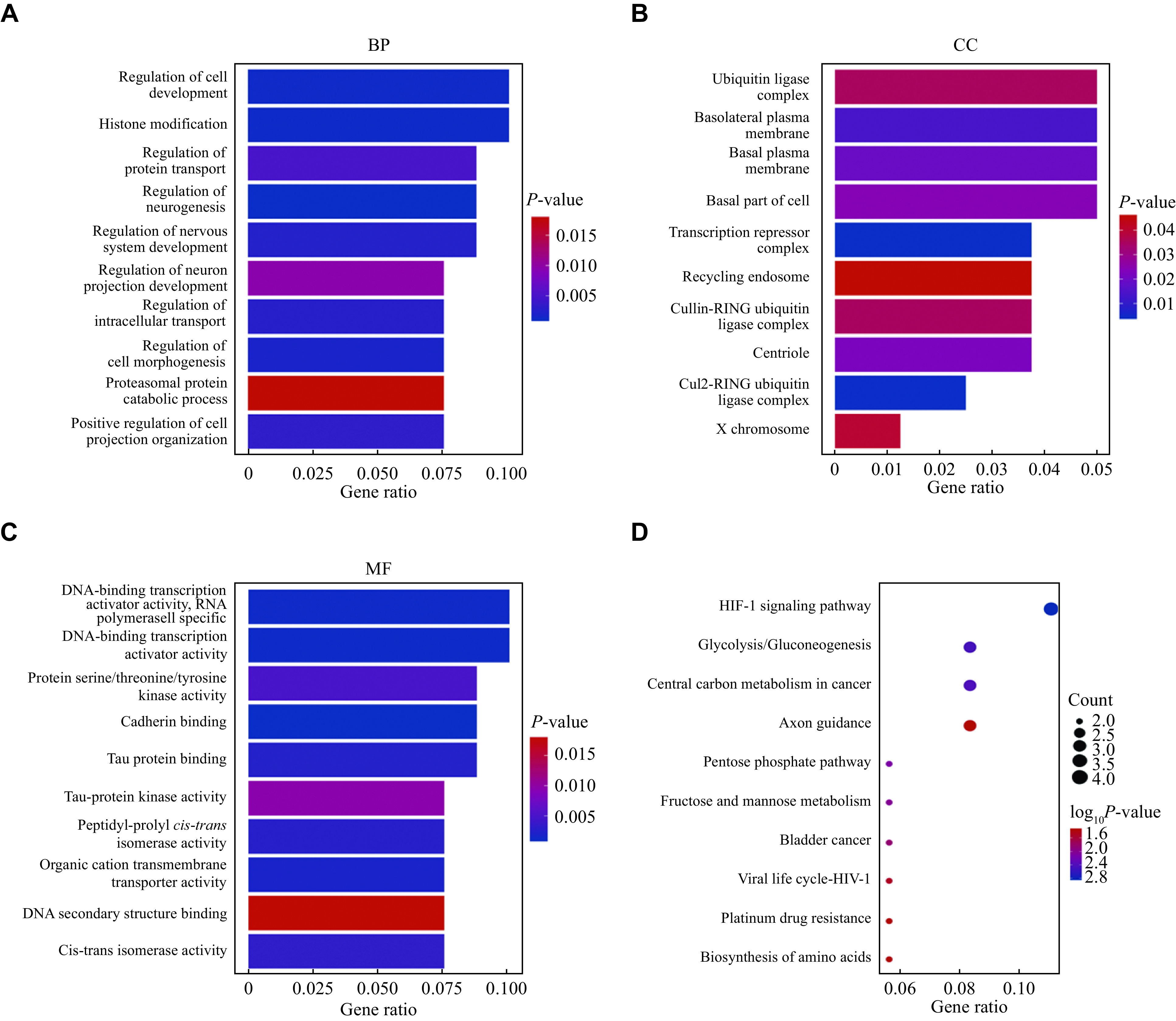
GO and KEGG enrichment of differentially expressed mRNAs in the competitive endogenous RNA network.

To determine the altered biological functions in the competitive endogenous RNA network, we applied KEGG and GO analyses. The top 10 GO terms in biological process (BP), cell component (CC), and molecular function (MF) were listed. In the BP section, histone modification, regulation of protein transport, regulation of cell morphogenesis, regulation of intracellular transport, positive regulation of cell projection organization, and proteasomal protein catabolic process were enriched. In terms of CC, genes located in the basolateral plasma membrane were most significantly altered. The obviously dysregulated MF was DNA-binding transcription activator activity (RNA polymerase Ⅱ-specific) ( ***Fig. 8A– 8C***). The enriched GO terms of BP, CC and MF categories for mRNAs in the competitive endogenous RNA network are listed in ***Supplementary Table 4*** (available online).

Apart from GO analysis, KEGG pathway enrichment analysis was performed, and the enriched pathways were ranked according to *P*-values. The top 10 dysregulated pathways were the hypoxia-inducible factor-1 (HIF-1) signaling pathway, glycolysis/gluconeogenesis, central carbon metabolism in cancer, axon guidance, pentose phosphate pathway, fructose and mannose metabolism, bladder cancer, viral life cycle-HIV-1, platinum drug resistance, and biosynthesis of amino acids ( ***Fig. 8D***). The results of KEGG pathway enrichment are listed in ***Supplementary Table 5*** (available online) **.**

### Validation of genes in the competitive endogenous RNA network by RT-qPCR

**Figure 9:**
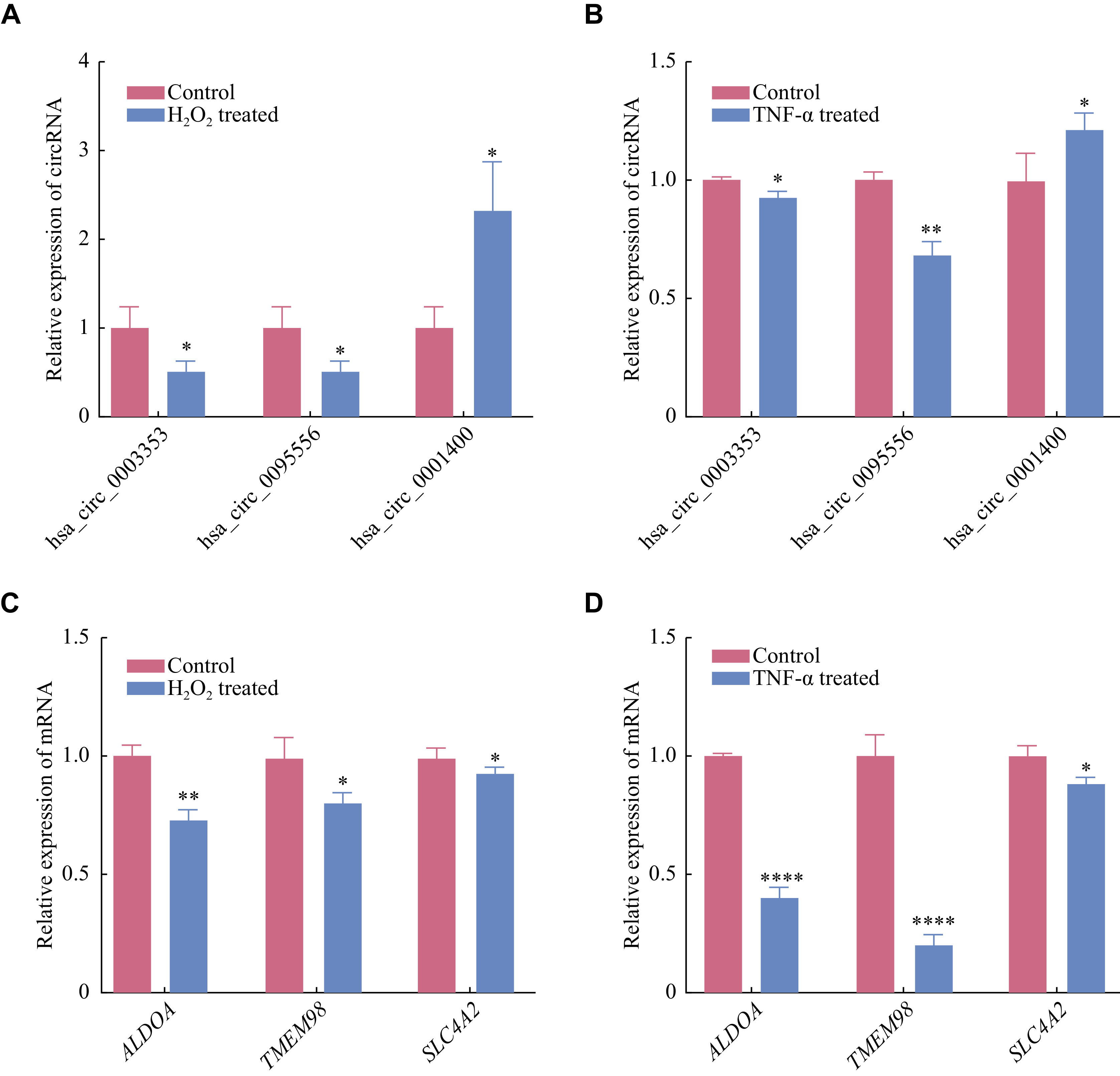
Validation of co-regulated circRNAs and mRNAs in the competitive endogenous RNA network.

We next performed RT-qPCR to verify the expressions of RNAs in the ceRNA network to confirm the sequencing results. Three cricRNAs (hsa_circ_0003353, hsa_circ_0095556, and hsa_circ_0001400) and three mRNAs ( *ALDOA*, *TMEM98*, and *SLC4A2*) were randomly selected from this network and subjected to PCR validation in the two cell models. In line with the RNA-seq data, hsa_circ_0001400 expression was upregulated in the two AMD models, while the expression of hsa_circ_0003353, hsa_circ_0095556, *ALDOA*, *TMEM98*, and *SLC4A2* were downregulated ( ***Fig. 9A***– ***9D***). These results further confirmed the RNA-seq results.

## Discussion

In the current study, we used TDM, a cross-platform integration method, to analyze three sequencing datasets (GSE29801, GSE50195, and GSE135092) from the GEO database. We used GSEA to discover new pathological processes, and PPI to show protein interaction. Based on the GSEA results, oxidative stress and TNF-α signaling *via* NF-κB ranked among the top 10 pathways and were chosen as disease models. After constructing the two cell models (oxidative stress and inflammation), circRNA expression profiling was performed. Altogether, we identified 83 upregulated and 86 downregulated circRNAs in the oxidative stress cell model as well as 39 upregulated and 25 downregulated circRNAs in the inflammation cell model. In the two cell models, six co-upregulated and eight co-downregulated circRNAs were chosen, and their biological functions were predicted by the competitive endogenous RNA network.

The current study used cross-platform normalization to combine independent transcriptomic datasets from AMD samples. Dataset combination provides a larger sample size and allows for a greater statistical power in identifying differentially expressed genes as well as pathological events. In addition, integrating data from different platforms can minimize confounding factors caused by the platform. Such integration is widely used in both tumor subtyping and the identification of differentially expressed genes ^[ 21] ^. TDM is a new cross-platform combination method developed to convert the distribution of data from RNA-seq to microarray. It performs better than other normalization methods in case of a small sample size ^[ 10] ^. We also used GSEA for bioinformatics analysis. GSEA has been developed to determine if a set of predefined genes are dysregulated in different phenotypes. It has a greater power than single gene analysis in detecting small but biologically significant changes in a series of genes. This method considers the whole transcriptomic data and can provide more information than GO and KEGG analyses. In contrast to the meta-analysis conducted by Dhirachaikulpanich *et al* ^[ 9] ^, we focused on dry AMD instead of various disease stages. Also, we applied a different combination method and used GSEA to reveal pathological events. Based on these differences, we observed differences in the revealed pathways. Another meta-analysis conducted by Saddala *et al* ^[ 22]^ explored differentially expressed genes in the periphery section of RPE/choroid/sclera samples. Interestingly, we both found altered complement activities in our samples, indicating that such a pathological process is fundamental across different parts of the fundus.

Based on these new bioinformatic methods, we identified a series of pathological events, including the increased activity of peroxisome, inflammation, mTORC1, cell glycolysis, and the decreased activity of complement. We revealed that the increased level of mTORC1 and glycolysis in RPE caused cell dedifferentiation and hypertrophy in disease states. Consistently, Zhao *et al* found that mitochondrial dysfunction triggered the AKT/mTOR pathway, causing an increase in glycolysis and photoreceptor degeneration ^[ 23] ^. Kurihara *et al* also found that the increased glycolysis in the presence of hypoxia leaded to AMD-like lesions in the RPE ^[ 24] ^. Although these pathways have previously been discovered in experimental studies, such changes have not been revealed in clinical samples. We believe that this is the first study to prove these two pathways through transcriptomic analysis.

The current study found a decreased complement activity in RPE/choroid. RPE/choroid is capable of producing complementary components and several complement regulators for immune surveillance, such as CD46, CD59, and complement factor H (CFH). CD46, a regulator of C3, is decreased in geographic atrophy, and its decrease can occur before identifiable AMD changes ^[ 25] ^. CD59 can inhibit the formation and fusion of C5b-9 (a membrane attack complex), protecting RPE/choroid from excessive complement activation. A decrease in CD59 labeling was found in the geographic atrophy area. For C5b-9 formation, the RPE and choroid must rely on systemic complement. These decreased complement regulators in RPE/choroid, along with the increased systemic C5b-9 levels, can trigger the pathology of AMD.

PPI analysis revealed that THBS2, LUM, and NR1H4 were the nodes with more than five connections. The upregulation of THBS2 is positively related to inflammation in nonalcoholic fatty liver disease and fibrosis ^[ 26] ^. While the increased inflammation is recognized by GSEA, *THBS2*, the hub gene demonstrated by PPI, further supported the conclusion at the protein levels. LUM was found to be up-regulated in the aqueous humor of patients with dry AMD ^[ 27] ^. Additionally, LUM can predict the clinical diagnosis of AMD. NR1H4 is a transcription factor that regulates lipid metabolism and suppresses inflammation ^[ 28] ^. As RPE cells undergo dysregulated lipid metabolism in AMD ^[ 29] ^, the downregulation of NR1H4 expression revealed by our data may be the key regulator of this disease.

Considering that AMD is a multifactorial disease triggered by RPE dysfunction, we built two cell models according to our bioinformatic analysis to simulate the disease status. To the best of our knowledge, this is the first study that constructed two disease models to verify the universality and reliability of the results. Among these co-regulated circRNAs, hsa_circ_0003353 expression was upregulated in the peripheral blood mononuclear cells from rheumatoid arthritis patients ^[ 30] ^. Mechanistically, it acted as a competitive endogenous RNA of miR-31-5p to activate immunity responses. Considering the down-regulation of hsa_circ_0003353 expression in the cell models, it may be protectively suppressed in the inflammatory cell model. Hsa_circ_0001400 served as a biomarker in multiple sclerosis, which is an autoimmune disease involving innate immunity ^[ 31] ^. Hsa_circ_0001658 elevated the level of oxidative stress in gastric cancer by suppressing autophagy ^[ 32] ^. Additionally, hsa_circ_0001658 expression was increased in dental pulp stem cells treated with TNF-α, which is a cell model of osteoarthritis ^[ 33] ^. The roles of co-regulated circRNAs in other diseases further confirmed their correlations with inflammation and oxidative stress.

Given that circRNAs can regulate biological processes by targeting miRNAs, we further constructed a competitive endogenous RNA network using online tools. Subsequently, 15 circRNA-miRNA pairs were predicted by binding sites. The biological functions of target genes in the competitive endogenous RNA network were further analyzed by GO and KEGG. The HIF-1 signaling pathway and glycolysis/gluconeogenesis were the most obviously changed pathways. The transcription factor HIF-1α is an oxygen-dependent transcriptional activator. Under normoxic conditions, its transcriptional activity is induced by growth factors, ROS, and inflammation ^[ 34] ^. ROS has been reported to mediate the stabilization, translocation, and activation of HIF-1α ^[ 35] ^. Inflammatory factors, including TNF-α, are reported to stabilize HIF-1α and promote its nuclear translocation, irrespectively of hypoxia. Based on these studies, it is plausible that HIF-1α is co-regulated by both pathological pathways. HIF-1α can regulate glucose transporters and glycolytic enzymes, which explains the second ranking of glycolysis/gluconeogenesis. These results show that HIF-1α is a common downstream target of inflammation and oxidative stress in RPE. A previous study also discovered that metabolic stress induced by either hypoxia or pseudohypoxia could lead to dry AMD-like pathologies ^[ 24] ^. Our bioinformatic results together with this experimental discovery can provide novel insights into the pathogenesis and progression of AMD. However, future studies are needed to understand the exact regulation role of circRNA in the HIF-1 signaling pathway.

The current study has several limitations. One is the lack of animal models, and clinical samples are also needed to reveal more convincing results *in vivo*. In addition, more experiments are needed to demonstrate the exact correlation between the competitive endogenous RNA network and biological functions of differentially expressed circRNAs. Moreover, circRNAs have more complex biological functions, such as protein translation, decoys, transporters, and protein scaffolds. Future studies are needed to explore these novel functions of circRNAs in AMD.

### Conclusions

In summary, cross-platform integration can help increasing statistical power in finding differentially expressed genes and pathological events. The circRNA profiles in cell models can help us to understand the molecular mechanisms participating in AMD. These circRNAs may play important roles in the disease, and as such, we believe future studies are needed to validate and prove their roles.
